# Diversity, Dynamics and Therapeutic Application of *Clostridioides difficile* Bacteriophages

**DOI:** 10.3390/v14122772

**Published:** 2022-12-12

**Authors:** Janet Y. Nale, Anisha M. Thanki, Srwa J. Rashid, Jinyu Shan, Gurinder K. Vinner, Ahmed S. A. Dowah, Jeffrey K. J. Cheng, Thomas Sicheritz-Pontén, Martha R. J. Clokie

**Affiliations:** 1 Centre for Epidemiology and Planetary Health, Department of Veterinary and Animal Science, Scotland’s Rural College, Inverness IV2 5NA, UK; 2 Department of Genetics and Genome Biology, University of Leicester, Leicester LE1 7RH, UK; 3 School of Pharmacy, University of Lincoln, Lincoln LN6 7TS, UK; 4 School of Life Sciences, University of Warwick, Coventry CV4 7AL, UK; 5 Center for Evolutionary Hologenomics, The Globe Institute, University of Copenhagen, 1353 Copenhagen, Denmark; 6 Centre of Excellence for Omics-Driven Computational Biodiscovery, AIMST University, Bedong 08100, Kedah, Malaysia

**Keywords:** *Clostridioides difficile*, *Clostridium difficile*, bacteriophages, phages, phage therapy, infection models

## Abstract

*Clostridioides difficile* causes antibiotic-induced diarrhoea and pseudomembranous colitis in humans and animals. Current conventional treatment relies solely on antibiotics, but *C. difficile* infection (CDI) cases remain persistently high with concomitant increased recurrence often due to the emergence of antibiotic-resistant strains. Antibiotics used in treatment also induce gut microbial imbalance; therefore, novel therapeutics with improved target specificity are being investigated. Bacteriophages (phages) kill bacteria with precision, hence are alternative therapeutics for the targeted eradication of the pathogen. Here, we review current progress in *C. difficile* phage research. We discuss tested strategies of isolating *C. difficile* phages directly, and via enrichment methods from various sample types and through antibiotic induction to mediate prophage release. We also summarise phenotypic phage data that reveal their morphological, genetic diversity, and various ways they impact their host physiology and pathogenicity during infection and lysogeny. Furthermore, we describe the therapeutic development of phages through efficacy testing in different in vitro, ex vivo and in vivo infection models. We also discuss genetic modification of phages to prevent horizontal gene transfer and improve lysis efficacy and formulation to enhance stability and delivery of the phages. The goal of this review is to provide a more in-depth understanding of *C. difficile* phages and theoretical and practical knowledge on pre-clinical, therapeutic evaluation of the safety and effectiveness of phage therapy for CDI.

## 1. Scope of Current Review and Introduction to *Clostridioides difficile* Infection 

There have been a number of reviews on *Clostridioides difficile* bacteriophages (phages) which summarise the mechanistic aspects that underpin our understanding and application of these phages [1,2,3,4]. In this review, we complement existing reviews by covering the body of data that has been largely gathered from our research and by others on a range of models developed to test the efficacy of *C. difficile* phages. We also emphasise the applied aspects of phages for phage product development.

*C. difficile* is a Gram-positive bacterium first isolated by Ivan Hall and Elizabeth O’Toole from the intestinal tract of infants where it was regarded as a commensal [5]. *C. difficile* infection (CDI) was later linked to antibiotic use and described as the cause of pseudomembranous colitis and nosocomial diarrhoea [6,7]. CDI is mediated by virulence factors located on a 19.6 kb pathogenicity locus (PaLoc), and the key toxins A and B are encoded by genes *tcdA* and *tcdB*, respectively [8,9]. Both toxins are cytotoxic, proinflammatory and cause disruption of tight junctions in human intestinal epithelial cells, resulting in fluid accumulation and damage to the large intestine. Expression of toxins A and B are controlled by the *tcdR* and *tcdC* genes, also located on the PaLoc [10,11]. Some strains, including the NAP1/027 epidemic strain, produce a third toxin, called the *C. difficile* binary toxin, which is located on the CdtLoc and may contribute to increased toxin production and disease severity [12,13]. The final toxin regulatory gene, *tcdE*, intercalates between toxins A and B, and is suspected of promoting the lysis of the cytoplasmic membrane and the release of the toxins from the cells [14]. Other virulence factors associated with *C. difficile* are linked to adhesion (such as pili, flagella, surface-layer proteins and physiological features), hydrolytic enzyme production, sporulation, biofilm production and cell wall glycopolymers [15,16,17,18,19,20].

Since the discovery of *C. difficile*, research has focused on virulence, pathogenicity and epidemiology of the bacterium to improve our understanding of CDI [21,22]. Despite advances made in the fields of antibiotic stewardship, infection control measures and surveillance policies, CDI remains a global health problem in the healthcare system [23,24,25,26,27]. The number of reported cases, recurrences and deaths is persistently high in many parts of the world [23,28]. In the UK, there are approximately twenty-two cases per 100,000 patient-bed days, in the EU, there are twenty-six infections in every 100,000 patient-bed days, and in the USA, there are one hundred and fifteen cases per 100,000 patient-bed days [16,23,24,29,30]. 

Strikingly, the infection has an associated ~45% recurrence rate and ~40% death rate [23,31]. The consequences of the infection are far reaching, affecting patient care and quality of life, and causing high economic costs; for example, in France, CDI’s annual costs are ~EUR 15 million [32]. These data highlight that CDI treatment and management strategies are insufficient, and that there is an unmet urgent need for alternative treatments to effectively treat the infection [4,22,31]. 

CDI is currently treated with the antibiotics fidaxomicin, vancomycin, and metronidazole [33,34]. New antibiotics such as tinidazole, rifaximin, rifalazil, and bezlotoxumab are currently being investigated and have some promise in treating CDI [31,35]. Antibiotics are, of course, essential, but bacteria are notorious for inducing gut dysbiosis, which triggers the outgrowth and colonisation of *C. difficile* and other pathogenic Enterobacteriaceae; hence, they are particularly problematic for CDI [36]. To overcome the low efficacy due to the development of antibiotic resistance and harmful side effects of antibiotic use in CDI, other therapies such as probiotics, immunotherapies, traditional and recombinant vaccines, monoclonal antibodies, faecal microbiota transfer, endolysins, and phage (a virus of bacteria) therapies are being developed as supplements or adjuncts to antibiotics [4,22,37]. 

Being natural and abundant organisms, phages are generally easy to isolate and characterise compared to the time and effort involved in developing many other therapies. Pertinent to CDI, phages have great advantages, as they can lyse bacteria with great precision to ensure the effective removal of the pathogen whilst maintaining other gut commensals, thus preventing dysbiosis [4]. Also, in the presence of susceptible bacterial strains, phages will continue to replicate and provide a continual supply of infective viral particles in the gut [38,39,40]. Importantly, phages are self-limiting, and thus are eliminated when the targeted bacteria have been cleared [41]. 

*C. difficile* can produce biofilms, which are aggregates of bacteria that adhere to surfaces, secrete protective extracellular polymeric substances, and can significantly impede antibiotic efficacy [42,43,44]. However, phages can effectively prevent the formation of *C. difficile* biofilms in vitro and penetrate mature biofilms to remove bacteria, which can potentially enable other therapeutics to access bacterial targets [45]. Clearly, these suitable properties of phages make them attractive and appropriate for CDI treatment, and this need has triggered several studies on their isolation and efficacy testing in infection models. We review and discuss the methods used to isolate and characterise the phages and the models we have developed and tested to assess phage lysis efficacy. We also discuss various ways *C. difficile* phages can be engineered and formulated to improve lysis, stability and therapeutic efficacy in humans and animals. 

## 2. *C. difficile* Phage Isolation and Characterisation

Phages are the most abundant biological entity on earth. They are widespread throughout all environments, and their presence is closely linked with their host bacteria. *C. difficile* phages were first isolated in the 1980s and initially used as a bacterial typing tool. However, their efficiency for typing was restricted as they could only infect a limited number of *C. difficile* strains, and also due to the fastidious nature of *C. difficile* itself [46,47]. These characteristics of *C. difficile* has also negatively impacted therapeutic research conducted on its associated phages [48]. However, the rapid rise in CDI incidence and severity due to antibiotic failures has triggered renewed interest in therapeutic phage development [2,4,49,50,51,52,53,54]. In this section, we focus on the different methods used for *C. difficile* phage isolation, identification and therapeutic application. 

### 2.1. Direct Isolation of Phages from Environmental and Clinical Samples

To begin with, we discuss direct screening of patient and animal faecal and environmental samples for the presence of infective *C. difficile* phages. For therapeutic purposes, strictly lytic phages, which infect and lyse target bacteria, are preferable to lysogenic phages that have the potential to integrate within the bacterial host chromosome. Lysogenic (or temperate) phages may cause horizontal gene transfer of genes associated with antimicrobial resistance (AMR) and other virulence factors associated with CDI [2,49,55]. The cycle the phage follows is identified via sequencing, and in genomes of lysogenic phages, genes associated with lysogeny, such as repressor and integrase genes, are found which are not present in lytic phages. Thus, attempts have been made by several research groups to screen for virulent lytic *C. difficile* phages. 

Metagenomic studies have revealed that the human gut has over 1000 species of bacteria and associated phages [56,57]. Furthermore, the high diversity and richness of phages in faecal samples of healthy humans is speculated to be the catalyst for the success of faecal microbiota transplantation [58,59]. However, despite clear evidence of diverse phages in healthy individuals and patients, strictly lytic *C. difficile* phages have not been observed. Also, directly isolating phages from human faecal samples, or indeed isolating strictly lytic phages from any environment, has been unsuccessful [60]. 

Whilst the reason for the lack of isolation of strictly virulent phages remains unknown, it may largely be linked to adaptation strategies, where *C. difficile* phages have evolved to exist alongside their hosts through lysogeny to enhance their survival in harsh environmental conditions. This is further supported as all characterised *C. difficile* genomes encode multiple prophages, an active clustered regularly interspaced short palindromic repeat (CRISPR) targeting phages, and the phages themselves encode CRISPR arrays that target additional phages [61,62,63,64]. This obligate interconnectedness with their hosts potentially limited the evolution, and thus existence, of strictly lytic *C. difficile* phages. Alternatively, *C. difficile* lytic phages may exist but are not amenable to existing isolation procedures for this organism.

To increase the possibility of isolating strictly lytic *C. difficile* phages, environmental and clinical samples have been enriched [60,64,65,66,67,68]. The enrichment method involves incubating environmental and clinical samples in liquid media inoculated with susceptible bacterial hosts to enable amplification of effective phages [65,66,69,70]. However, this approach may limit the diversity of prospective phages observed due to bias towards the strain(s) included [70]. The enrichment media may also be supplemented with antibiotics to select for *C. difficile* growth and proliferation, reduce competition by other bacterial species and to allow optimum amplification of phages to occur [64,67]. Salts (MgCl_2_ and/or CaCl_2_) can also be added to the enrichment mix to enhance the stability and attachment of the putative phages present in the samples to the bacterial hosts [66,68]. However, studies enriching faecal samples have only observed *C difficile* phages in ~10% of samples examined and none from sewage [66], despite examining large numbers of a wide variety of samples, including those from healthy humans, inflammatory bowel disease patients and from healthy pigs, as well as pig caecal contents and slurries [60,65]. Sources from which *C. difficile* phages have been isolated include soil, sediment and estuarine samples, but all of these locations may be associated with human activities and, hence, could suggest why phages were isolated [64,67]. 

### 2.2. Isolation of C. difficile Phages through Prophage Induction

We stated in the previous section that *C. difficile* phages can be isolated from clinical and environmental samples through enrichment procedures. However, all *difficile* phages isolated to date are lysogenic and encode integrases in their genomes; this is despite their clear-plaque morphology, often broad host range (they can infect multiple *C. difficile* strains) and lysis ability determined using various in vitro, ex vivo and in vivo model systems [53,71,72,73,74]. Clearly, the presence of lysogeny-associated genes in the genomes of *C. difficile* phages indicates that they are temperate despite them behaving in a lytic manner. Furthermore, in some cases, lysogens have been isolated from the interaction between the phages and their bacterial hosts, signifying that the integrases are active [53,75]. 

In cases where strictly lytic phages that target species such as *C. difficile* cannot be found, it is pragmatic to isolate phages that infect pathogenic strains of interest and to assess their therapeutic potential. There is still lots to learn about phage lifestyles even if all integrases are active. There is a possibility that the integrases observed in some *C. difficile* phage genomes are only active within a subset of specific strains rather than the strains being examined. Certainly, our work has shown that in some cases, despite subjecting the strains to very high concentrations of phages, lysogens were not formed [53]. Therefore, if the phages are effective, research can be conducted to assess the risks associated with using temperate phages in their native state for therapeutic purposes. If temperate phage genes such as integrases pose a risk of horizontal gene transfer and will therefore fail to meet the regulatory standard, then the phage could potentially be genetically modified to delete all temperate associated genes [76]. 

To provide a solution to the problem of *C. difficile* phage isolation, we developed a method to induce prophages (lysogenic phages) from *C. difficile* strains. We previously hypothesised that prophage induction from environmental *C. difficile* strains might be effective to isolate therapeutically relevant phages that can lyse clinically relevant *C. difficile* strains [77,78]. To do this, bacterial strains are treated with sub-lethal concentrations of various DNA-damaging agents to mediate prophage release (Table 1). In *E. coli*, this exposure was shown to trigger the *recA* pathway and the SOS response, which resulted in the cleavage of prophage(s) from the host chromosome [79]. The released phages are recovered by centrifuging and filtering the cultures. The lytic activity of induced phages can be confirmed by spot tests or plaque assay techniques [80,81]. 

Two DNA-damaging agents, mitomycin C (0.3–5 ug/mL final concentrations) and irradiation with UV light (302 nm wavelength), are commonly used to induce prophages in *C. difficile*. Mitomycin C is an alkylating agent that initiates DNA damage by causing mispairing of bases, DNA strand damage or cross-linking of complementary strands as shown in *E. coli* [79,82]. Although mitomycin C is widely used, norfloxacin (a fluoroquinolone) was found to enhance prophage induction, especially in strains not susceptible to induction by mitomycin C [78,83]. This may be attributed to the mechanism of norfloxacin action, which inactivates the DNA gyrase and topoisomerase IV, causing the disruption of DNA supercoiling that leads to damage [84]. There are no standardised procedures that guide the selection of the prophage-inducing agent in *C. difficile*, but studies have shown that the use of diverse agents on one strain could maximise prophage release and yield [78]. Regardless of the inducing agent used, to maximise yield, prophage induction has been carried out at different growth phases of the bacterial broth culture [78,83]. Although lysis and reduction of bacterial growth are generally considered to be good indicators of prophage release, we have also observed that the treated bacterial cultures often continued to grow or remained stationary despite phage release [78]. 

## 3. Diversity of *C. difficile* Phages

### 3.1. Morphological Diversity of C. difficile Phages 

Isolated *C. difficile* phages to date belong to the Caudovirales family, which is the order of tailed phages (Table 1) [1,2,4,85,86]. Over the past decade, the phage taxonomy has been updated, and currently there is a new order called Tubulavirales along with ten new families [87]. However, as published data on *C. difficile* phages was based on the previous taxonomy, we will refer to these phages using the old taxonomy scheme for consistency. 

There are thirty-five *C. difficile* phage genomes publicly available to date and all have dsDNA genomes. Twenty-six of the phages were classified as myoviruses, eight were siphoviruses and one is a phage tail-like protein (Table 1). The isolated myoviruses have been further sub-classified based on their tail lengths, which are the medium-tailed and short-tailed myoviruses [85]. No podovirus that targets *C. difficile* has been isolated [1]. The phage tail-like particles, also referred to as bacteriocins (or diffocins), lack a capsid and are widely isolated from various *C. difficile* strains either alone or simultaneously in addition to other phage morphologies [78,83]. Although the particles have been shown to have bactericidal ability, they were not able to replicate effectively to form plaques [83]. 

### 3.2. Genomic Diversity

Due to the highly diverse nature of phage genomes, there are no generalised conserved genes to characterise them as seen in the bacteria using the 16S rRNA gene [77,78]. However, specific *C. difficile* phage genes have been identified that could be used as molecular markers to examine diversity [77,78]. The major capsid protein is relatively conserved and has been used to identify *C. difficile* prophages in situ [78]. However, this marker is limited as it is too diverse and not recognisable in some phages, such as in phage phiCD27. Similarly, the minor capsid protein, gp20, is also too diverse to facilitate alignment and primer assertions [78]. Due to these limitations, the holin gene, which is identifiable in all phages, has been used to assess genetic diversity within *C. difficile* phages [77]. Although the holin is also limited due to its conserved nature, it is useful in distinguishing between siphoviruses and myoviruses that infect *C. difficile*. 

**Figure 1:**
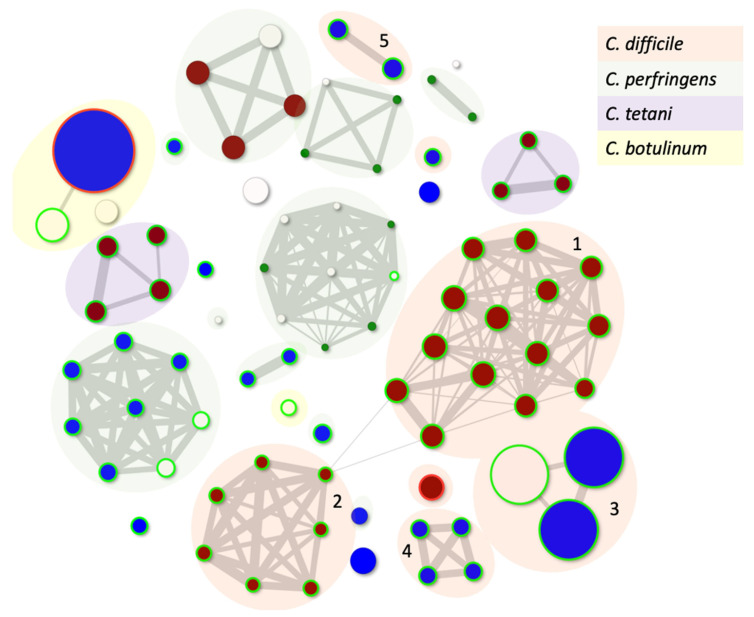
Genetic relationships between all known Clostridial phages that can either independently plaque on bacterial hosts or can be induced from their host strain. The size of the dot is related to the genome size, and the colour is reflective of the morphology with red being a myovirus, blue siphovirus and white where the taxonomy is unknown. The green circle suggests the phages are temperate using Phage Leads, and the red circle shows that the phage carries an antibiotic resistance gene. Clouds numbered 1–5 represent clusters of *C. difficile* phages (Table S1). Phages included in the analysis are listed in Table S1.

The diversity of *C. difficile* phage genes and modularity within their genomes were described recently [1]. However, to both contextualise and understand the genetic relationships and genetic diversity within *C. difficile* and all Clostridial phages, we have applied our PhageClouds concept [88]. PhageClouds is a computational database, and the concept was developed for better visualisation and understanding of the relationships between phages that target any bacterium of choice [88]. This approach is based on creating phage genomic networks from whole genome similarities and thereby overcomes the limitations imposed by only examining one conserved gene. PhageClouds allows us to identify phages that are most closely genetically related to each other, here represented as particular clusters or clouds (Figure 1, Table S1). Where any phages share DNA, they group together, and we will see that the clouds are connected through those genetic similarities. On the other hand, different clouds of phages which are not connected do not have any DNA similarities. 

Figure 1 shows the relationship between all known Clostridial phages. It is clear that there is no genetic relationship between the phages that infect *C. difficile* and other Clostridial species, as they form different clouds. There are five distinct groupings/clouds of *C. difficile* phages, although the major and largest two, clouds 1-2 (containing twenty-one and nine phages, respectively), are clearly connected. Interestingly, these clouds represent the myoviruses that target *C. difficile* (Table S1). The third cloud contains a group of eight relatively newly described related siphoviruses with genomes that are much larger than most *C. difficile* phages, approximately 133 kb (*Clostridioides* phage LIBA-2945, *Clostridioides* phage LIBA-6276, *Clostridium* phage phiCD211) [89,90]. These phages have not yet been shown to propagate using the lytic life cycle but can be induced from the genomes of their hosts and have intriguingly long tails [90]. The fourth cloud consists of the remaining *C. difficile* siphoviruses (*Clostridium*_phage_phiCD6356, *Clostridium*_phage_phiCD24-1, Clostridium_phage_phiCDKH01, *Clostridium*_phage CPD2), which are clearly genetically distinct from each other, and from all other phages sequenced to date, and thus appear on this figure as pairs or singletons. 

## 4. Phage Mechanics of Infection

### 4.1. Impact of Tail Fibres on Attachment and Host Selection

**Figure 2:**
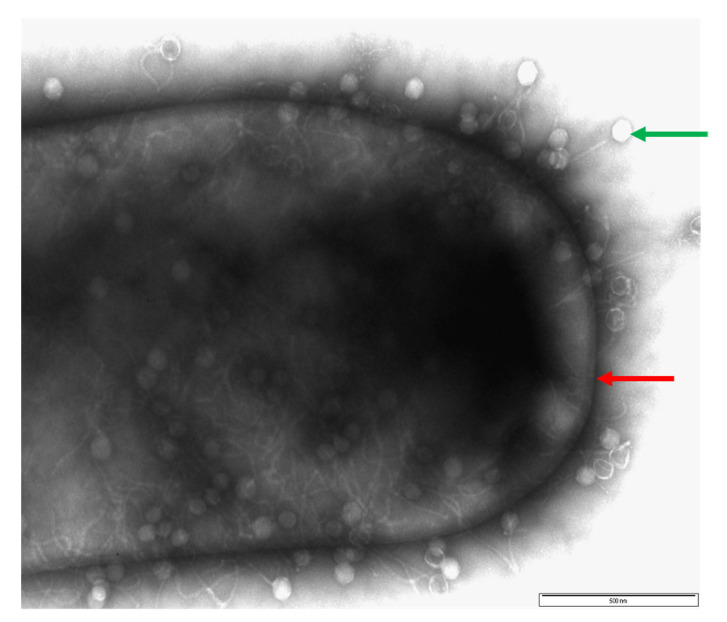
Image of a CD105LC1 *C. difficile* cell (red arrow) surrounded by phage phiCDHS1 (green arrow), which have attached to the surface of the bacterial cell wall. Exponentially growing bacteria cultures were mixed with phage at a ratio of 1:100 (bacteria to phage ratio) and allowed to attach for 15 min. Afterward, the phage/bacterial cultures were fixed with 2% glutaraldehyde and mounted on glow-discharged pioloform/carbon-coated copper grids for 5 min. After being washed with water, samples were stained with uranyl acetate, air-dried and examined using a JEOL 1220 electron microscope at 80 kV voltage. Bar is approximately 500 nm.

The first stage of a successful phage infection is attachment to specific receptors on the bacterial host cell as shown in Figure 2 [88]. Phage binding occurs through several interactions between the receptor binding proteins (RBPs) via two stages. The first stage involves the phage tail fibres reversibly attaching to a receptor on the surface of the bacterial cell, and the second involves the irreversible attachment to the same receptor or a different receptor. The phage then injects its genetic material into the host cells [91]. Extensive research has focused on unraveling these mechanisms in Gram-negative bacteria, in particular *E. coli* phages, as both the organism and its phages are easier to mutate and handle in the laboratory [92]. In comparison, Gram-positive bacteria such as *C. difficile* are more difficult to work with in the laboratory, largely due to their fastidious nature and cell wall composition, which make them difficult to manipulate. However as there is growing interest in studying the mechanism of infection of phages targeting Gram-positive bacteria, methods to understand this interaction are being developed, and the phage receptors in *Bacillus subtilis, Lactococcus lactis* and *Staphylococcus aureus* have been identified through mutational studies [93,94,95,96]. Whilst for *C. difficile* phages the mechanisms of action by which phages infect their hosts are unknown, the methods developed for Gram-positive bacteria may be applied. 

However, our research group has been making progress in understanding phage–host interactions, and we investigated adsorption of *C. difficile* phages both to strains they do and strains they do not infect. Our study included three myoviruses (phiCDHM1, phiCDHM3 and phiCDHM6), and we identified phages phiCDHM1 and phiCDHM3 bound by ~75% to strains they infect and by less than 30% to strains they do not infect. However, phage phiCDHM6 adsorbed to all strains by ~30% regardless of whether or not it could infect the strain, despite the tail-fibre proteins of phiCDHM3 and phiCDHM6 sharing 100% homology at the amino-acid level. Thus, phage adsorption is phage–host specific, and our study provided insights into phage infection [97]. Currently, the phage receptors on *C. difficile* are unknown, however, we speculate that *C. difficile* phages could attach to the surface layer (S-layer) proteins on *C. difficile* cells, as phage tail-like bacteriocins were shown to use S-layer proteins as their receptors [94]. 

### 4.2. Phage Host Range

Successful phage binding of virulent phages leads to infection and the lysis of bacteria to release progeny. The range of available bacterial species or strains a phage can lyse is known as its host range, and phages that lyse multiple strains from different subgroups of the same bacterial species are more clinically useful for therapy [98]. In comparison, some phages have narrow host ranges and can only lyse one strain from a single subgroup [99]. To maximise efficacy, phage cocktails can be used which include a diverse set of phages which target different strains and thus can improve overall lysis efficiency [100]. To further improve phage efficacy and specificity, the RBPs and phage tail-fibre proteins could be genetically engineered, as they are involved in phage specificity. Resultant modified phages can therefore recognise, attach and lyse a broader set of host targets, and the method has been successfully shown in *Pseudomonas aeruginosa* and *Acinetobacter baumannii* phages [101]. 

### 4.3. Impact of Phage Infection on C. difficile Physiology

Analysis of phage genomes has highlighted *C. difficile* phage-encoded genes that can mediate transcriptional regulations in the bacterium. For example, *C. difficile* phage phiCDHM1 encodes the *agr* system, consisting of a cassette of genes (*agrA, agrB, agrC* and *agrD*) with the ability to modulate how the bacterium interacts with the environment. These genes impact bacterial motility, biofilm formation, defence, toxicity, replication, metabolism, sporulation, stress response and quorum sensing [3,74,102]. In addition, phage phiCD119 has been shown to modulate toxin production after lysogenisation [75]. 

To further understand the impact of phage infection on *C. difficile*, we recently conducted a transcriptional study investigating infection of phage phiCDHS-1 on *C. difficile* strain R20291 to determine which genes are expressed during infection. The analysis revealed that 10–20% of the bacterial host genes are differentially expressed during infection [103]. The majority of these genes were downregulated at the early stage of the phage life cycle, which includes genes responsible for metabolism and DNA replication [103]. A similar study of R20291 infection by phage CD38-2 showed that genes associated with transcriptional regulators and phosphotransferase system subunits involved in glucose, fructose, and glucitol/sorbitol uptake and metabolism were differentially expressed in the host. Other differentially expressed host genes were linked to phase variation regulated by the conserved phase-variable cell-wall protein [104]. Also, genes responsible for lysis–lysogeny decision were expressed at an early infection stage of *C. difficile* phage infection [103,105]. Furthermore, various genes related to pathogenicity, such as toxin production and regulation, anti-phage systems, bacterial sporulation and adhesion, were all regulated during phage infection [103,105]. Interestingly, though phage infection resulted in bacterial resistance and lysogeny development, the clones produced were less virulent, further supporting the use of *C. difficile* phage for therapeutic purposes, as will be discussed in the next section [103]. 

## 5. Therapeutic Application of *C. difficile* Phage Models of *C. difficile* Phage Therapy 

Infection leading to lysis is the key phage asset, which can be harnessed for phage therapy and has been studied using in vitro, in vivo and ex vivo models [47,52,53,100,106]. In this section, we discuss the different models developed and used to study the lytic activity of *C. difficile* phages. 

### 5.1. Culture-Based Assays 

Several in vitro studies have been conducted to ascertain the efficacy of *C. difficile* phages to either kill or reduce bacteria using both host-range and virulence assays [45,52,53,106]. Host-range analysis is typically conducted by applying specific volumes of high-titre phage stocks on confluent cultures of *C. difficile* in semi-solid media, and the same phage is tested on multiple clinically relevant *C. difficile* strains which represent different ribotypes. Host range analysis has identified *C. difficile* phages to have narrow-to-broad host ranges, often lysing several ribotypes [53,64,65]. We used this method to screen the host range of our seven phages against 80 strains, representing 21 clinically relevant ribotypes from humans. We identified phiCDHM4 as having the narrowest host range, and lysed single representative strains from each of four ribotypes. In comparison, phages phiCDHM3, phiCDHS-1, and phiCDHM5 had broad host ranges and infected 20–31 strains representing 10–12 ribotypes. However, the results of host-range analysis showed complimentary coverage could be achieved by combining the phages as cocktails; for example, phage cocktail phiCDHM1+2+5+6 combined is able to lyse 18 ribotypes and 62 of the strains tested [53]. 

In addition to host-range analysis, killing or virulence assays are used to determine which phages or phage cocktail combinations are efficient at lysing target strains [50]. This method involves growing the target strain to an exponential stage and then infecting it with phage(s) and monitoring bacterial growth over a set time, typically 24 h. We used this method to identify optimal phage combinations for lysis of *C. difficile*, and we tested two-, three- and four-phage cocktails [53]. We found the three-phage cocktail, phiCDHM2+5+6, caused a 6 log10 reduction in bacterial counts over 24 h, whilst the four-phage cocktail, phiCDHM1+2+5+6, was more efficient and lysed the same culture within 3 h (0 log10) [53]. In addition, with the four-phage cocktail there was no regrowth of *C. difficile* over 24 h. As the four-phage cocktail was more efficacious, it could be a potential candidate for future phage clinical trials. 

### 5.2. Biofilm Model

As discussed in the introduction, *C. difficile* strains can aggregate in complex biofilms in vitro, and these structures complicate therapeutic deployment of antibiotics and act as reservoirs for recurrent CDI [44,45,107,108,109,110]. Unlike antibiotics, data from our study showed that *C. difficile* phages can inhibit biofilm formation by penetrating and lysing established biofilms, which leads to a decrease in bacterial viability and biomass [45]. Furthermore, the four-phage cocktail we have developed, phiCDHM1+2+5+6, was more effective than using single phages at tackling biofilms and could be an assuring therapeutic option for controlling *C. difficile* biofilms [45].

### 5.3. Epithelial Cell Tissue Model

Human cell lines are informative ex vivo tools to study phage/bacterial interactions and therapeutic efficacy. We examined the interaction of the *C. difficile* 027 strain with phage phiCDHS-1 in the presence of two human epithelial cell lines [111]. The cell lines Caco-2 and HT-29 were selected, as both have previously been used to study the pathogenesis of *C. difficile* [112,113,114,115,116]. The data from the study revealed that pre-treatment of cell cultures with phiCDHS-1 one hour prior to introducing *C. difficile* significantly reduced *C. difficile* counts from 8 log10 to 3 log10 CFU/mL within eight hours. In comparison, by introducing phiCDHS-1 and *C. difficile* simultaneously to the epithelial cells, *C. difficile* counts were reduced from 8 log10 to 4 log10 CFU/mL within the same treatment time. There was established evidence that the phage was able to adsorb to the epithelial cells, which may have contributed to the effectiveness of the prophylactic treatment [111]. 

### 5.4. Batch Fermentation Model 

In vitro gut and batch fermentation models simulate the human gut microbiome and have been developed as a useful tool to study the gut microbiome response to anti-infectives, including phages [52,106,117,118,119]. The main goal in using the fermentation model is to culture a complex intestinal microbiota to study microbial modulation and metabolism under controlled environmental conditions. This approach is both time-efficient and cost-effective compared to animal models [52,106,118,119,120]. 

One study used an in vitro batch fermentation model spiked with faecal material from a single donor [106,119]. Single-phage treatment with phiCD27 led to a substantial decrease in vegetative *C. difficile* cells numbers, low toxin level detection and no detrimental impact on human gut commensals [106,119]. Building on this model, we tested the effectiveness of our optimised four-phage cocktail, phiCDHM1+2+5+6, to clear *C. difficile* in a fermentation vessel inoculated with combined faecal slurries from four individuals from different ethnicity and age groups [52]. The phage cocktail efficiently cleared *C. difficile* from the model within 24 h, and *C. difficile* was not recovered. Phage prophylactic treatment was more effective than remedial treatment, consistent with previous data [52,106,119]. Encouragingly, in addition to preserving the gut commensals, phage treatment enhanced the colonisation of specific commensals, further strengthening their use in preventing dysbiosis and CDI [52].

### 5.5. Galleria Mellonella Infection Model 

The use of *G. mellonella* as a bacterial infection model has risen in popularity within the last decade, ranging from simple survival assays to multifaceted experiments. The larval stage of the greater wax moth is used as a favorable ethical, financial and experimental ease model compared to other models [121,122]. *G. mellonella* is predominately used as a screening model to assess virulence of a particular bacterium or gene. The survival outcome and melanization of larvae during infection provide a macro-view of host–infection outcome, whilst changes in larval gene expression and proteomic responses have been measured to provide a more precise insight into infection [51,123,124]. The larval response to bacterial infection and toxic substances is similar to other commonly used cell lines and models [125]. Some caveats still exist, however, as *G. mellonella* larvae only possess an innate immune response, which, although sharing similarities to the mammalian humoral and cellular responses, lacks the complexity of mammalian-based models [126,127]. The lack of adaptive immune response, however, can be useful to study solely for the interactions between pathogen-innate immunity. 

Before using this model to explore CDI phage therapy, we first established colonisation of the *G. mellonella* larvae with *C. difficile* using oral inoculation rather than the hemolymph for better reproducibility. Having established this model, we then tested the efficacy of phage cocktail phiCDHM1+2+5+6 to reduce *C. difficile* colonisation and improve the survival of challenged larvae. Three phage treatment regimens were tested: prophylactic (phage inoculation prior to bacterial infection); concurrent (simultaneous bacterial and phage infection); and remedial (phage treatment after bacterial infection). Prophylactic phage treatment was the most effective treatment, and 100% of larvae survived after 60 h. In comparison, there was a 0% survival rate of larvae infected only with *C. difficile*. Phage treatment also reduced *C. difficile* counts to 2 log10 CFU/larva, whilst in larva infected only with *C. difficile*, counts were 8 log10 CFU/larva [45]. This observation of prophylactic treatment being the most effective concurred with the biofilm data and with the cell tissue culture assays [45,111]. 

We were able to further refine the *G. mellonella* CDI model by measuring insect stress genes as biomarkers to detect and monitor disease progression and recuperation during phage therapy in the insects [51,103]. This approach allowed an increased resolution into determining the phage cocktail efficacy and other potential antimicrobial agents [51,123]. Such advancements in the development of the *G. mellonella* infection model provide an attractive alternative to more conventional approaches to studying CDI and might provide a valuable tool to track infection for other pathogens.

### 5.6. Hamster Infection Model

The study of phage therapy within in vitro models usually provides the preliminary groundwork in pre-clinical studies, allowing more experimental control and traceability without raising ethical complications. However, the linearity of such parameters limits the complexity or representability of assessment when compared to an in vivo model, where a systemic approach provides additional dimensions of interaction, such as an immune system, microbiota or even confounding variables [128]. Hamster models (especially Golden Syrian hamsters) have been the predominant choice to study CDI, sharing similarities with antibiotic-induced susceptibility and clinical manifestations observed in humans [129,130]. As a result, they are ideal candidates for phage therapy studies and efficacy testing of different cocktails. 

The first reported phage therapy in a CDI-induced in vivo study was conducted by Ramesh et al. where hamsters were subjected to clindamycin-induced CDI and treated with various doses of phage CD140 [100]. Untreated hamsters were susceptible to CDI within 72 h, displaying diarrhoea and haemorrhagic and fluid-filled ceca, while all but one phage-treated hamster survived [100]. *C. difficile* was recovered from all culled hamsters and, interestingly, the strain recovered from them was resistant to phage CD140. The emphasis on the fleeting protection of phages were highlighted, as CD140 was not recovered from the cecal contents from the hamsters fourteen days after phage therapy. Additionally, through clindamycin-induced *C. difficile* reinfection fourteen days after phage therapy, all hamsters succumbed to *C. difficile* rechallenge of the same strain, further enforcing the temporal nature of phage therapy. The ability for hamsters to pick up environmental phages was observed, as 50% of the control group exhibited phage recovery, which was attributed to colonisation through phage-contaminated environments previously used to house phage-treated hamsters [100]. As a by-product of their study into in vivo phage lysogenisation with PCR, Govind et al. demonstrated an increased survival rate of phage-treated hamsters of 5 days, whereas the non-treated controls died within 48 h [75].

The assessment of phage therapy for CDI in vivo is still in its infancy; the use of phage cocktails in the hamster CDI model has only been reported from our laboratory. Different combinations of *C. difficile* phages were analysed first in vitro, and the five most promising combinations were assessed in the clindamycin-induced hamster colitis for up to five days [53]. The role of phage treatment in *C. difficile* colonisation was assessed through bacterial recovery from luminal and tissue samples. The most effective treatment was determined to be the four-phage combination (phiCDHM1+2+5+6), which was able to reduce recovered bacterial load in the lumen samples by 4 log10 CFU/g and tissue samples by 2 log10 CFU/g. Furthermore, this combination of phage intervention promoted increased survivability in hamsters by approximately ~32 h compared to untreated controls [53].

## 6. Genetic Manipulation of *C. difficile* Phages

Over the last fifteen years, significant progress has been made in developing tools to mutate *C. difficile* [131]. Researchers have faced numerous hurdles, as gene transfer in *C. difficile* is less efficient, and developing stable mutants in *C. difficile* has been a consistent problem due to the lack of counter-selection markers [132]. However, tools are now available to produce stable mutations in *C. difficile*, which can be applied to mutate *C. difficile* phages in their lysogenic state, but no genetic tools are currently available to mutate lytic phages [2]. This section will describe the three main *C. difficile* genetic manipulation systems, the ClosTron system, *Clostridium* shuttle plasmids and CRISPR, which are all tools that can be used to genetically mutate *C. difficile* phages [133]. Of particular interest would be the ability to delete the lysogeny-associated genes in phages, which will likely pose a problem for their downstream therapeutic applications [53].

### 6.1. ClosTron System

The ClosTron system uses broad-host-range group II introns for directed mutagenesis within Clostridia. Group II introns are described as catalytic RNAs that can excise themselves from RNA transcripts and then insert themselves into a new target [134]. The mobility of group II introns provides a method for gene disruption, and the intron target specificity can be altered by changing the DNA sequence that encodes the section of the intron. The group II introns carry an open reading frame in which they have a multifunctional intron-encoded protein (IEP) [135]. Through the action of the IEP, the introns are able to self-catalytically splice out of the targeted RNA sequence in the host gene. The IEP synthesises the corresponding complementary DNA strand via activity of reverse transcriptase [134]. The ClosTron system also includes an integrated functional antibiotic resistance gene within the coding region of the group II intron element; thereby, the acquisition of antibiotic resistance is coupled with integration and helps to positively select for integration events [136,137]. 

The directed mutagenesis process involves four clear steps: step 1 is intron design; step 2 is plasmid construction, both of which are done using an easy-to-follow online tool (http://clostron.com (accessed on 28 October 2022)); step 3 is plasmid transfer via conjugation; and step 4 is integrant isolation, which is performed using standard methods [136]. The ClosTron system has been successfully used for directed mutagenesis in *C. difficile* and has helped to improve our knowledge on adhesion and virulence genes, and genes involved in dissemination of *C. difficile* [138,139,140]. Similarly, the system can be used to mutate and study temperate *C. difficile* phages.

### 6.2. Clostridium Shuttle Plasmids

Other tools developed for direct mutagenesis via allele exchange include a range of pMTL8000 *Clostridium*-*Escherichia coli* modular shuttle plasmids [141]. Allele exchange occurs when the native allele of DNA is exchanged with a new allele that contains a mutation by homologous recombination [142]. The shuttle plasmids in the pMTL8000 range are modular and all include a Gram-negative replicon, an antibiotic selection marker, a Gram-positive replicon, a conjugal transfer function and/or a multiple cloning site [130]. The plasmids contain a Gram-negative replicon, which allows all the cloning to be initially done in *E. coli*, i.e., genes of interest and flanking regions added to the plasmid in *E. coli*, as cloning directly into *C. difficile* is difficult. Then the plasmid can be transferred to *Clostridium* via conjugation [143]. However, it should be noted that DNA transfer to *C. difficile* occurs at low frequencies and varies significantly between *C. difficile* strains. Furthermore, the conjugation efficiency is dependent on the length of the flanking regions used and the media used for the conjugation process [142].

The pMTL8000 plasmids are replication-defective plasmids (pseudosuicide) and are used for allele exchange via a two-step recombination process. The first recombination event involves the integration of the plasmid into the target genome and is referred to as a single crossover event. Strains in which the first recombination event has occurred grow rapidly on selective media, and their colonies are larger in size, which makes them easier to identify [131]. The second recombination event is the plasmid excision from the genome, at which point cells can either revert to wild-type or generate mutants [142]; however, the frequency of the second recombination event occurring to generate double-crossover mutants is low and very laborious [144]. To overcome this problem, a counter selection marker (toxic under certain conditions), the *codA* gene of *E. coli*, has been identified and used successfully to generate double-crossover mutants at a higher frequency [132].

The shuttle plasmids were used by our group to tag *C. difficile* phages in their lysogenic state with luminescence *luxAB* genes (reporter phages) with the aim of developing a phage-based diagnostic test. To design the reporter phages, non-essential phage genes were replaced with the *luxAB* genes, which would then be expressed during phage infection. We found this method required extensive optimisation, and once the reporter phages had successfully been constructed, the *luxAB* genes were unstable within the phage genome and were lost during phage replication. We are further optimising the method to develop stable mutations in *C. difficile* phages [145]. 

### 6.3. CRISPR Technology

The CRISPR system is an RNA-mediated immune system in prokaryotic cells, and the type II CRISPR-Cas9 has been widely used to genetically modify both eukaryotic and prokaryotic cells. In the CRISPR-Cas9 system, the Cas9 is directed by guide RNA (gRNA) to the region of chromosomal DNA in which to make the desired mutation, which then subsequently leads to breakage of the double-stranded DNA [146]. The system allows generation of stable mutants on the host chromosome and has been used to mutate numerous lytic and lysogenic phages of important pathogens including *Bacillus subtilis*, *Vibrio Cholerae* and *E. coli* 0157:H7 [147,148,149,150]. To date, the system has not been used to mutate *C. difficile* phages, but in the past three years, CRISPR-Cas9 plasmids have been designed to further study genes in *C. difficile*, which could potentially be applied to its associated phages [151].

The methods described in this section can be applied to temperate *C. difficile* phages, but future tools need to focus on developing methods to mutate lytic phages. For example, the lytic T7 *E. coli* phage was mutated using CRISPR–Cas3 technology. Phages were first propagated on a *E. coli* strain that harboured a plasmid, with the donor DNA and the phage sequences that flank either side of the gene to be deleted. Phage recombinants were enriched by plating on another *E. coli* strain that contained three plasmids that encode for CRISPR-Cas3 activity and the spacer sequence, which is complementary to the target gene. Phage recombinants were isolated at a rate of approximately 40% [152]. A simpler method was used to engineer a *Lactococcus lactis* phage P2, whereby the plasmid encoding CRISPR-Cas9 and the plasmid-encoded donor DNA were added to one strain. The authors showed that the method could be used to introduce insertion, deletion and point mutation in several sites in the genome of phage P2 [153]. It may be difficult to use similar CRISPR-based methods to mutate *C. difficile* phages, as it is currently difficult to transfer just one plasmid to *C. difficile*. Furthermore, there are no data to support stability of two or three plasmids in the same strain. Therefore, moving forward, a plasmid that encodes for CRISPR activity and includes the donor DNA is needed.

## 7. Encapsulation and Formulation of Therapeutic *C. difficile* Phages

Isolation, characterisation and purification of phages form the initial stages in the roadmap for commercialisation of therapeutic phages. It is equally important to evaluate their compatibility with commercialisation processes, including formulation, scale-up, storage and delivery. Success stories are often hindered by the lack of consideration to the post-processes and therefore should be evaluated alongside the first stage [154,155]. 

Formulating phage for delivery involves many challenges, since conventional off-the-shelf solutions are not suitable. Active pharmaceutical ingredients (APIs) are compatible with a catalogue of formulation ingredients and have been studied for decades; in the case of phages, however, there is limited knowledge. The first article discussing phage formulation was published in 2002 using the model *Salmonella*-specific phage, Felix O1. The authors encapsulated the phage in alginate and chitosan particles for oral delivery [156]. Since then, there has been a steady rise in the number of publications addressing the challenges around phage encapsulation and formulation.

Phages are prone to environmental changes such as temperature, humidity, pH and mechanical shearing, which makes them challenging to encapsulate and protect for therapeutic applications [157,158,159,160,161,162,163]. Apart from formulating phages in compatible materials, the technique used for encapsulation can prove detrimental to the viability of phages. Hence, the choice of encapsulating technique plays a crucial role in the development of commercially viable phage products. 

Spray-drying is a well-known industrial method for the encapsulation of API into dry powder form, which is easier to transport in comparison to liquids and gels. The method uses high temperatures (50–300 °C) to form aerosolized droplets which undergo evaporation, leaving fine particles. It has also been employed for the encapsulation of phages due to its ease of use and one-step process for obtaining a dry powder carrying phages [157,158,164,165,166]. In all instances, there has been reported losses of viability of phage ranging from 0.5–2 log10 PFU/g attributed to the high operating temperatures. However, phage loss can be limited by testing different encapsulation materials and adding sugars such as trehalose, which can protect phages during the drying process.

*C. difficile* phages were encapsulated in a methyl-methacrylate copolymer known as Eudragit S100, which is responsive to changes in pH [167]. Here, Eudragit was used in combination with alginate to produce composite microparticles using microfluidic technology, which enables precise control over size and monodispersity of the microparticles without the need for shear or high temperature. The microparticles were able to protect the phage from pH 2 (SGF: simulated gastric fluid) for a period of 3 h. A total loss of 1 log10 PFU/mL was observed during this period, resulting in the final phage titre of 5 log10 PFU/mL. The results demonstrated that the combination of the formulation and the technique employed to produce microparticles helped minimise the loss of phage post SGF exposure. Encapsulating phages using this method will enable successful transit of the phage to the site of infection in the lower GI tract, where *C. difficile* colonises in humans [167]. 

In addition to the above encapsulation techniques, there are a plethora of combinations which can be tested to find the optimal method and material for phage encapsulation. A more advanced method which offers further benefits in protecting phages and ensuring a site-specific release is lipid nanoparticles [168]. These can further be functionalised and combined with polymeric materials to achieve a desired release profile. Further work is still required in this field; however, with the continued success of the encapsulation of mRNA and other biologics, it gives hope to the future of phage encapsulation.

## 8. Conclusions

Research on *C. difficile* phages has progressed significantly over the past decade, from phage isolation, to sequencing and understanding phage-host interactions. In addition, robust in vitro, ex vivo and in vivo models have been developed to test efficacy of phages, and the collective data highlights that phages are effective against *C. difficile*. The barrier we face is that all isolated phages are temperate, and thus with current regulation may not be ideal for therapy. However, with the progression of genetic tools, we will be able to mutate or delete undesirable genes and progress the therapeutic use of phages against *C. difficile*. 

## Figures and Tables

**Table 1:** List of fully characterised, publicly available *C. difficile* phage genomes, their sources and methods of isolation.

Phage Name	Morphology	Isolation Method	Source	Accession Number
phiCDHS-1	Siphoviridae	Enrichment	Estuarine	KU057941
CDHM19	Myoviridae			NC_028996
CDHM11	Myoviridae			NC_029001
CDKM15	Myoviridae		Sediment	KX228400
CDKM9	Myoviridae			KX228399
phiMMP02	Myoviridae		Patients	NC_019421.1
PhiMMP03	Myoviridae			NC_028959
PhiMMP01	Myoviridae			NC_028883
PhiMMP04	Myoviridae			NC_019422
PhiCD418	Myoviridae		Sewage	MW512573
PhiCD2301	Myoviridae			MW512571
PhCD08011	Myoviridae			MW512572
PhiCD1801	Myoviridae			MW512570
PhiCD146	Siphoviridae	Induction (Mitomycin C)	UV 302 nm	NC_028958
PhiCD111	Siphoviridae			NC_028905
PhiCD 24-1	Siphoviridae			LN681534
PhiCD505	Myoviridae			NC_028764
PhiCD481-1	Myoviridae			NC_028951
PhiCD506	Myoviridae			NC_028838
PhiCD38-2	Siphoviridae		0.5–5	NC_015568
PhiCD6356	Siphoviridae			NC_015262
PhiCD27	Myoviridae			NC_011398.1
phiSemix9P1	Myoviridae			KX905163.1
phiCDHM1	Myoviridae			NC_024144
PhiC2	Myoviridae			NC_009231.1
CDHM13	Myoviridae			NC_029116
CDHM14	Myoviridae			LK985321
phiCDKH01	Siphoviridae			JACSDL010000003.1
JD032	Myoviridae			MK473382
HMC114	Phage tail-like particles			CM000660.1
LIBA6276	Siphoviridae		Unknown	MF547662.1
ΦCD1801	Myoviridae	Enrichment	Sewage	MW512570
ΦCD08011	Myoviridae			MW512572
ΦCD418	Myoviridae			MW512573
ΦCD2301	Myoviridae			MW512571

## Supplementary Materials

The following supporting information can be downloaded at: www.mdpi.com/article/10.3390/v14122772/s1, Table S1: Phages included in the phage cloud analysis shown in Figure 1.
