# Supplementary material for: Diversity, Dynamics and Therapeutic Application of Clostridioides difficile Bacteriophages
Source: Viruses. 2022 Dec 12;14(12):2772. doi: 10.3390/v14122772 (PMC9784644; doi:10.3390/v14122772)
Supplement: Supplementary file 1 [file viruses-14-02772-s001.zip › viruses-2030476-supplementary.pdf]

Table S1. Phages included in the phage cloud analysis shown in Figure 1.

	name	accession	cloud
**1**	Clostridium phage phiMMP02	JX145341	1
**2**	Clostridium phage phiMMP04	JX145342	1
**3**	Clostridium phage phiMMP01	LN681541	1
**4**	Clostridium phage phiCDHM14	LK985321	1
**5**	Clostridium phage phiCD505	LN681539	1
**6**	Clostridium phage CDKM9	KX228399	1
**7**	Clostridium phage CDKM15	KX228400	1
**8**	Clostridium virus phiCD27	EU719189	1
**9**	Clostridium virus phiCD27 NC	011398	1
**10**	Clostridium phage phiMMP03	LN681542	1
**11**	Clostridium virus phiCD119	AY855346	1
**12**	Clostridium virus phiCD119 NC	007917	1
**13**	Clostridium phage phiCDHM19	LK985322	1
**14**	Clostridium phage CDMH1	HG531805	1
**15**	Clostridium virus phiC2	DQ466086	1
**16**	Clostridium virus phiC2 NC	009231	1
**17**	Clostridium phage phiCD506	LN681540	1
**18**	Clostridium phage phiCDHM13	HG796225	1
**19**	Clostridium phage JD032	MK473382	1
**20**	Clostridium phage phiCD481-1	LN681538	1
**21**	Clostridium phage phiCDHM11	HG798901	1
**22**	Clostridium phage phiZP2	JQ729992	2
**23**	Clostridium phage CPQ7	MZ401007	2
**24**	Clostridium phage CPS2	MH248069	2
**25**	Clostridium phage CPQ1	MW057920	2
**26**	Clostridium phage phiCP7R	JQ729990	2
**27**	Clostridium phage CPQ9	MZ401008	2
**28**	Clostridium phage CPQ10	MZ401009	2
**29**	Clostridium phage CPQ3	MZ401005	2
**30**	Clostridium phage phiCPV4	JQ729991	2
**31**	Clostridium phage phiCP26F	GQ443085	3
**32**	Clostridium phage phiCP34O	JF767209	3
**33**	Clostridium phage phiCP39-O	EU588980	3
**34**	Clostridium phage vB CpeP PMQ04	MZ995505	3
**35**	Clostridium phage phiCP9O	JF767210	3
**36**	Clostridium phage phiCP13O	JF767208	3
**37**	Clostridium phage CPQ4	MZ401006	3
**38**	Clostridium phage CPD1	MH999280	3
**39**	Clostridium phage CPD4	MK017819	4
**40**	Clostridium phage CP3	MF001357	4
**41**	Clostridium phage CPAS-15	MN417334	4

**42**	Clostridium phage Clo-PEP-1	KY206887	4
**43**	Clostridium phage phiCT9441A	KM983329	5
**44**	Clostridium phage phiCT19406A	KM983330	5
**45**	Clostridium phage phiCT453A	KM983327	5
**46**	Clostridium phage phiCTC2A	KM983333	5
**47**	Clostridium phage phiCD111	LN681535	6
**48**	Clostridium phage phiCD146	LN681536	6
**49**	Clostridium phage CDSH1	KU057941	6
**50**	Clostridium phage phiCD38-2	HM568888	6
**51**	Clostridium phage CPS1	KY996523	7
**52**	Clostridium phage vB CpeP HN02	MW815121	7
**53**	Clostridium phage CPD2	MH999279	7
**54**	Clostridium phage phi24R	JN800508	7
**55**	Clostridium phage phiCD211	LN681537	8
**56**	Clostridioides phage LIBA2945	MF547663	8
**57**	Clostridioides phage LIBA6276	MF547662	8
**58**	Clostridium phage phiCT453B	KM983328	9
**59**	Clostridium phage phiCTC2B	KM983334	9
**60**	Clostridium phage phiCT19406B	KM983331	9
**61**	Clostridium phage c-st	AP008983	10
**62**	Clostridium phage D-1873	ACSJ01000000	10
**63**	Clostridium phage phiCD24-1	LN681534	11
**64**	Clostridium phage phiCDKH01	MN718463	11
**65**	Clostridium phage phi3626 NC	003524	12
**66**	Clostridium phage phi3626	AY082070	12
**67**	Clostridium phage susfortuna	MH393889	13
**68**	Clostridium phage CPD7	MK017820	13
**69**	Clostridium phage vB CpeS-CP51	KC237729	14
**70**	Clostridium phage phiCT19406C	KM983332	15
**71**	Clostridium phage PhiS63	JQ660954	16
**72**	Clostridium phage phiSM101	CP000315	17
**73**	Clostridium phage phiCTP1	HM159959	18
**74**	Clostridium phage HM T	KU517658	19
**75**	Clostridium phage CWou-2020a	CP063964	20
**76**	Clostridium phage CpV1	HM640230	21
**77**	Clostridium phage HM2	LT600745	22
**78**	Clostridium phage phi8074-B1	JQ246028	23
**79**	Prokaryotic dsDNA virus sp	MK892507	24
**80**	Clostridium phage phiCD6356	GU949551	25
**81**	Clostridioides phage phiSemix9P1	KX905163	26
**82**	Clostridium phage CWou-2020b	CP063968	27
